# Crystal structure of 1-amino-3-(4-chloro­phen­yl)-2-cyano-3*H*-benzo[4,5]thia­zolo[3,2-*a*]pyridine-4-carboxamide

**DOI:** 10.1107/S2056989025001562

**Published:** 2025-03-04

**Authors:** Nadia H. Metwally, Galal H. Elgemeie, El-shimaa S. M. Abd Al-latif, Peter G. Jones

**Affiliations:** a Chemistry Department, Faculty of Science, Cairo University, Giza, Egypt; b Chemistry Department, Faculty of Science, Helwan University, Cairo, Egypt; c Institut für Anorganische und Analytische Chemie, Technische Universität Braunschweig, Hagenring 30, D-38106 Braunschweig, Germany; Universität Greifswald, Germany

**Keywords:** benzo­thia­zole, hydrogen bonds, halogen bonds, crystal structure

## Abstract

In the structure of the title compound, C_19_H_13_ClN_4_OS, the four atoms of the pyridinic ring that are not fused with the thia­zole, including the *sp*^3^ C atom, lie significantly outside the benzo­thia­zole plane. A short intra­molecular S⋯O contact of 2.5992 (4) Å is observed. The amide NH_2_ group is planar, whereas the amine NH_2_ group is pyramidalized. The three-dimensional packing involves two inter­connected layer structures. The first, parallel to the *bc* plane, involves three classical hydrogen bonds N—H_amine_⋯O (one of two), N—H_amine_⋯Cl and one N—H_amide_ ⋯N_cyano_; the second, parallel to the *ab* plane, involves two hydrogen bonds, N—H_amide_⋯O and the second N—H_amine_⋯O, together with the short and linear contact N_cyano_⋯Cl—C, which may be regarded as a halogen bond.

## Abstract 2

The tricyclic ring system of the title compound departs from planarity in the region of the *sp*^3^ carbon atom. The three-dimensional packing involves four classical hydrogen bonds and one N⋯Cl halogen bond.

## 1. Chemical context

Benzo­thia­zole and its fused-ring derivatives are among the most important heterocyclic compounds used in medicinal chemistry and are essential constituents of many medicines and natural heterocyclic compounds (Ammazzalorso *et al.*, 2020 ▸). Fused benzo­thia­zoles have a variety of established pharmacological qualities that are useful in the search for new and important therapeutic medications (Wang *et al.*, 2009 ▸). Benzo­thia­zoles display noteworthy biological actions, including anti­bacterial (Kashyap *et al.*, 2023 ▸), anti­viral (Ke *et al.*, 2013 ▸) and anti­cancer (Irfan *et al.*, 2020 ▸) effects, and are thus significant compounds for drug development (Rana *et al.*, 2008 ▸); for some ongoing studies and associated discoveries, see Abdallah *et al.*, 2023*a* ▸,*b* ▸. The use of medications derived from benzo­thia­zole derivatives has been extensively developed in clinical practice to treat a range of illnesses with great thera­peutic efficacy (Huang *et al.*, 2009 ▸).

We are inter­ested in developing syntheses for the production of heterocycles based on benzo­thia­zoles (and other heterocycles) that may find application in medicine (Mohamed-Ezzat *et al.* 2024 ▸); in this respect, we have reported the biological activity of a range of 2-pyrimidyl- and 2-pyridyl benzo­thia­zole compounds with promising cytotoxic action (Azzam *et al.*, 2020*a* ▸,*b* ▸, 2022*a* ▸,*b* ▸).

**Figure 1:**
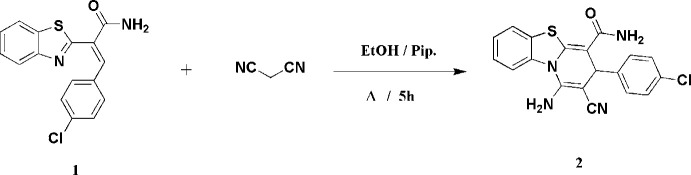
The synthesis of compound **2** (Pip. = piperidine).

As an extension of these results and our earlier studies (Metwally *et al.*, 2022*a* ▸,*b* ▸), the goal of the current study was to design and produce benzo­thiazo­pyridines. The title compound **2**, a substituted benzo[4,5]thia­zolo[3,2-*a*]pyridine-4-carboxamide, was synthesized in good yield by reacting 2-(1,3-benzo­thia­zol-2-yl)-3-(4-chloro­phen­yl)prop-2-enamide **1** with malono­nitrile in refluxing ethanol containing catalytic amounts of piperidine for 5 h (Fig. 1 ▸). We postulate that the reaction proceeds *via* the formation of Michael inter­mediate adducts. Compound **2** was previously synthesized by us using the reaction of 1,3-benzo­thia­zole-2-acetamide with 4-chloro­benzyl­idenemalono­nitrile (Fathy & Elgemeie, 1988 ▸). The crystal structure of **2** was determined to establish its structure unambiguously.
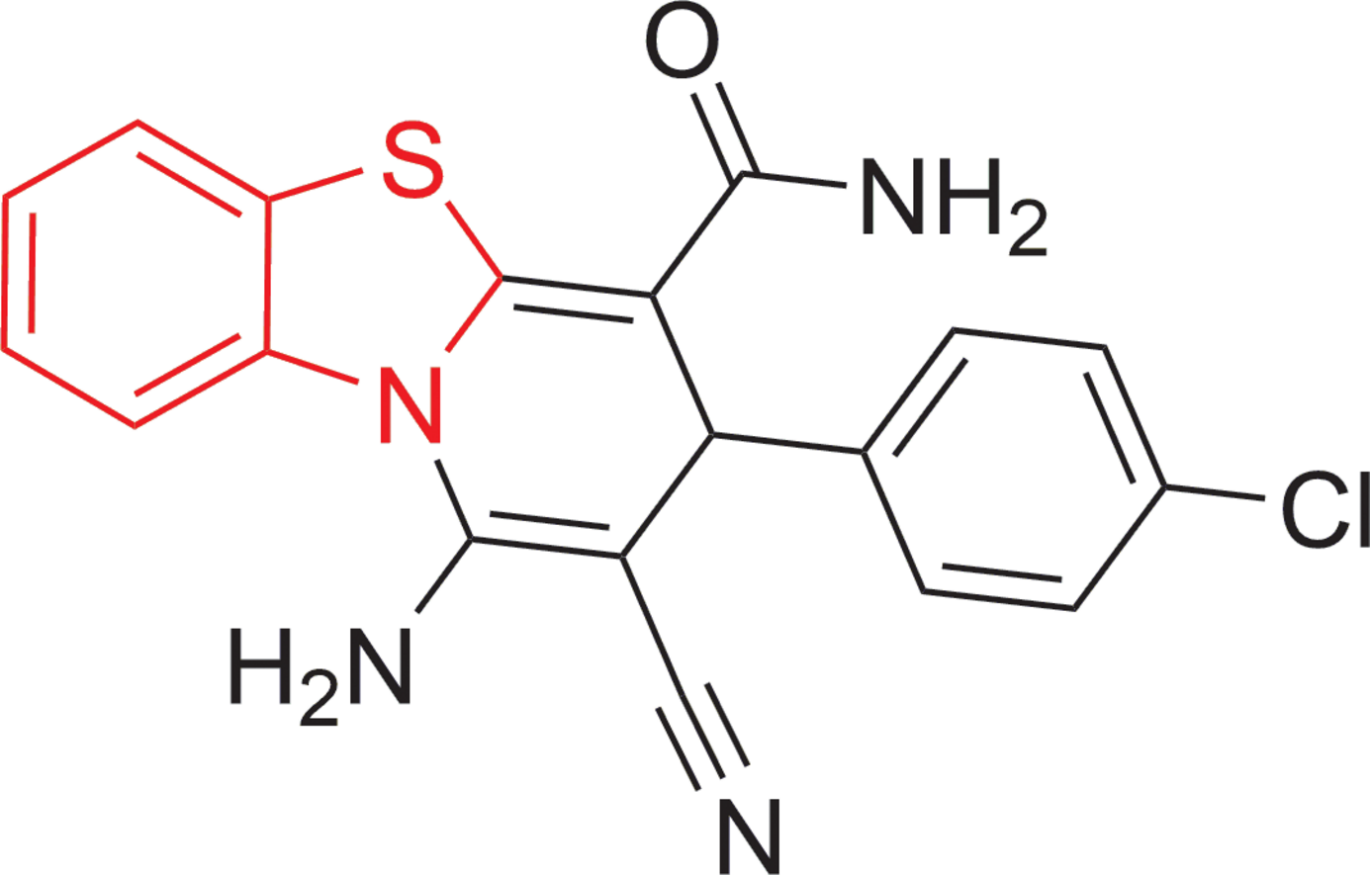


## 2. Structural commentary

**Figure 2:**
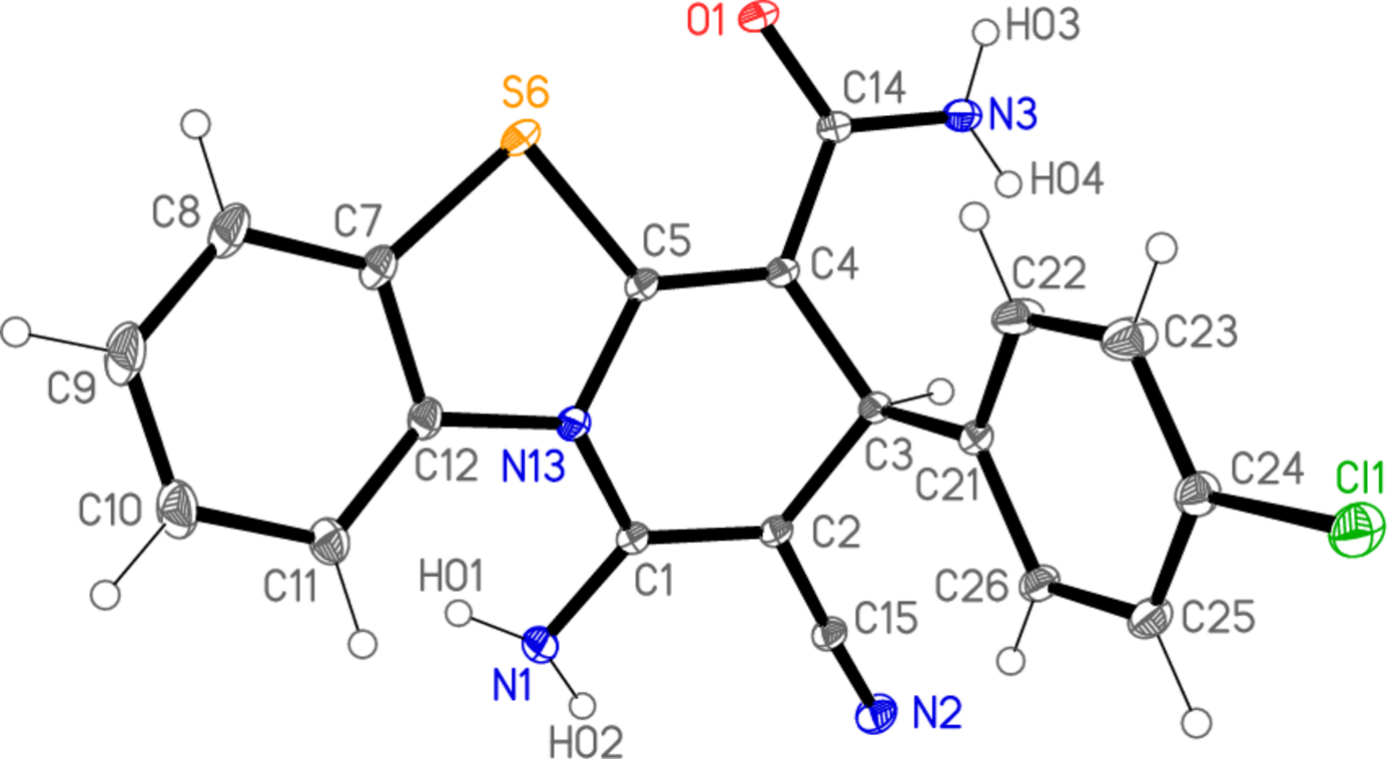
The mol­ecule of compound **2** in the crystal. Ellipsoids represent 50% probability levels.

**Figure 3:**
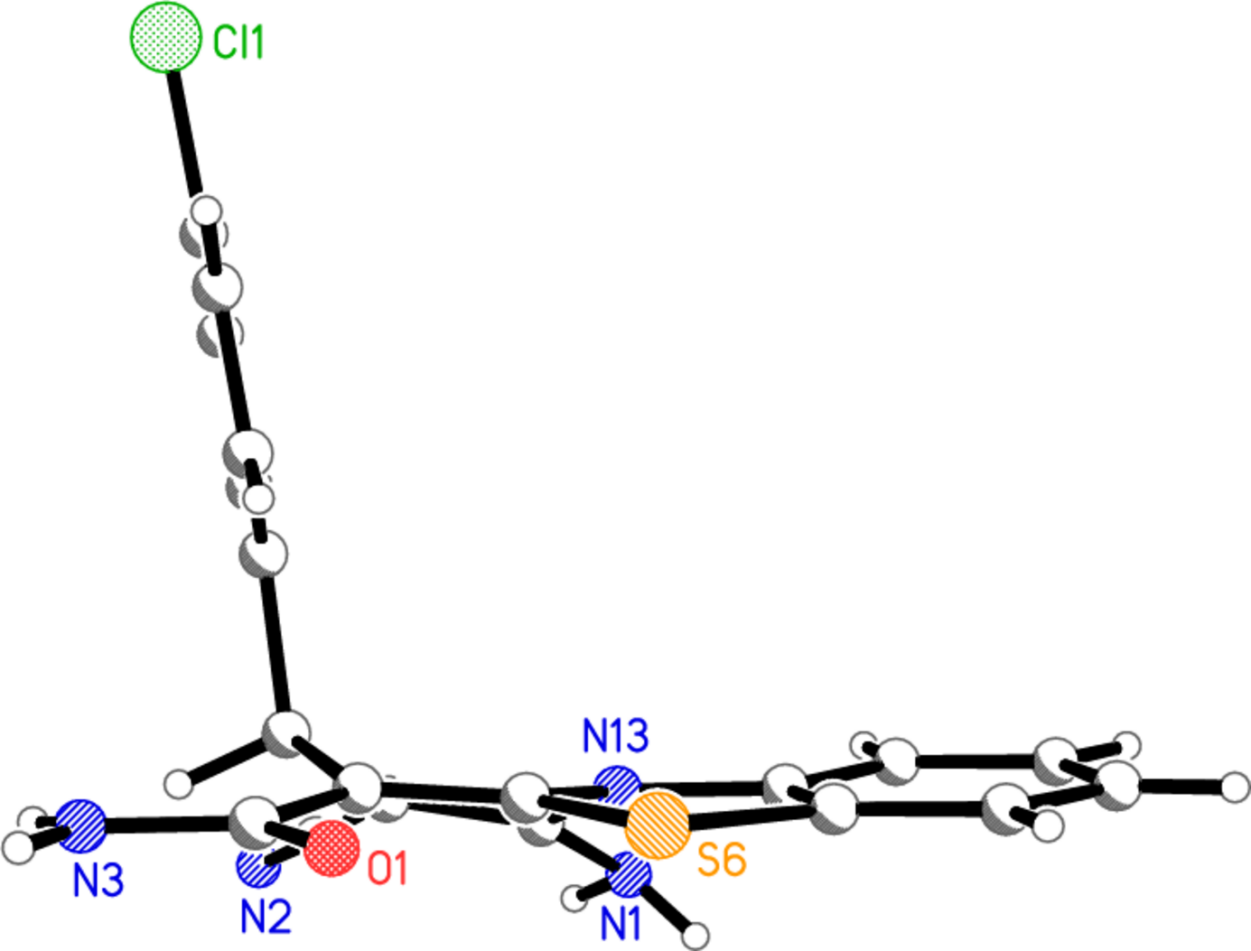
Side-on view of mol­ecule **2**.

The structure of compound **2** is shown in Fig. 2 ▸, with selected mol­ecular dimensions in Table 1 ▸. Bond lengths and angles may be considered normal, *e.g.* the wide formally *sp*^2^ external angles of *ca*. 125° at the junctions of five- and six-membered rings, and the bond lengths and angles around the *sp*^3^ atom C3. In the tricyclic ring system, the nine atoms C5–N13 are approximately coplanar (r.m.s. deviation = 0.04 Å); atoms C1, C2, C3 and C4 lie outside this plane by −0.474 (1), −0.234 (1), 0.492 (1) and 0.188 (1) Å, respectively. In the pyridinic ring, the atoms N13, C1, C2 and C3 are coplanar (r.m.s. deviation = 0.004 Å), with C4 and C5 lying outside this plane by 0.711 (1) and 0.556 (1) Å, respectively. The torsion angle C4—C5—N13—C1 differs markedly from zero, which may be associated with steric pressure imposed by the substituents at C1 and C4; however, N13, with its three at least formally single N—C bonds (*cf*. bond lengths in Table 1 ▸), may not be extensively involved in the aromatic system and thus would not necessarily impose planarity on the sequence C4—C5—N13—C1. The chloro­phenyl ring is approximately perpendicular to the grouping C5–N13 [inter­planar angle = 81.60 (1)°]; this is made clear by the side-on view of the mol­ecule in Fig. 3 ▸. The geometry of the nitro­gen atom N3 of the amide NH_2_ group is essentially planar (angle sum = 359.5°), whereas that at the amine nitro­gen N1 is pyramidalized (angle sum = 342.9°) and at N13 slightly pyramidalized (358.1°). There is a short intra­molecular contact S6⋯O1 of 2.5992 (4) Å that determines the orientation of the amide group, being associated with a synperiplanar geometry in the atom sequence S6—C5—C4—C14—O1.

## 3. Supra­molecular features

The mol­ecular packing is dominated by four classical hydrogen bonds from the hydrogen atoms of the NH_2_ groups (Table 2 ▸), together with the short contact N2⋯Cl1(1 + *x*, −1 + *y*, *z*) of 3.1296 (5) Å. The angle C24—Cl1⋯N2′ is 177.86 (2)°, and the linearity indicates that the inter­action is probably to be regarded as a halogen bond (see *e.g.* Metrangelo *et al.*, 2008 ▸).

**Figure 4:**
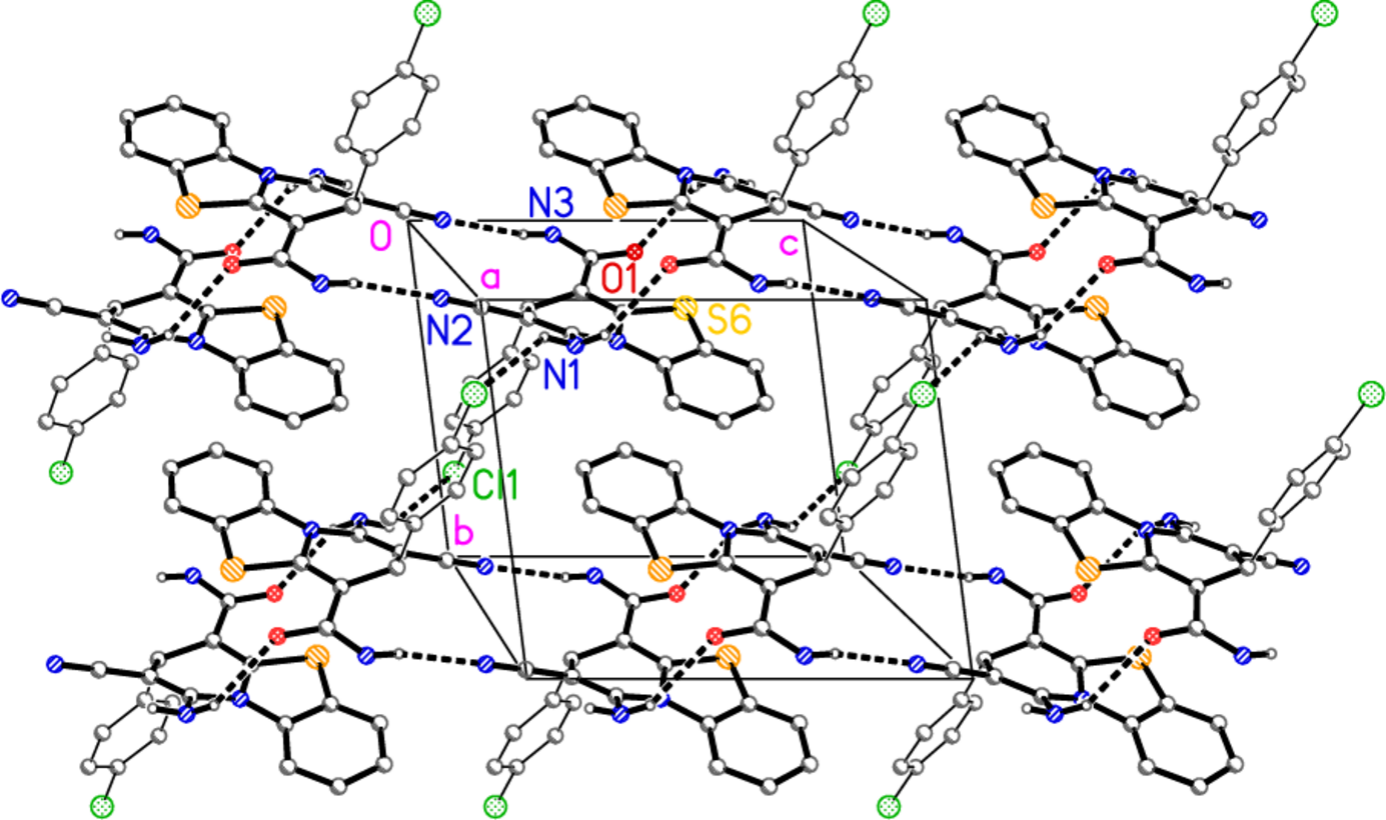
Packing diagram of compound **2** viewed perpendicular to the *bc* plane. Hydrogen bonds are indicated by thick dashed lines. Hydrogen atoms not involved in hydrogen bonds are omitted for clarity.

**Figure 5:**
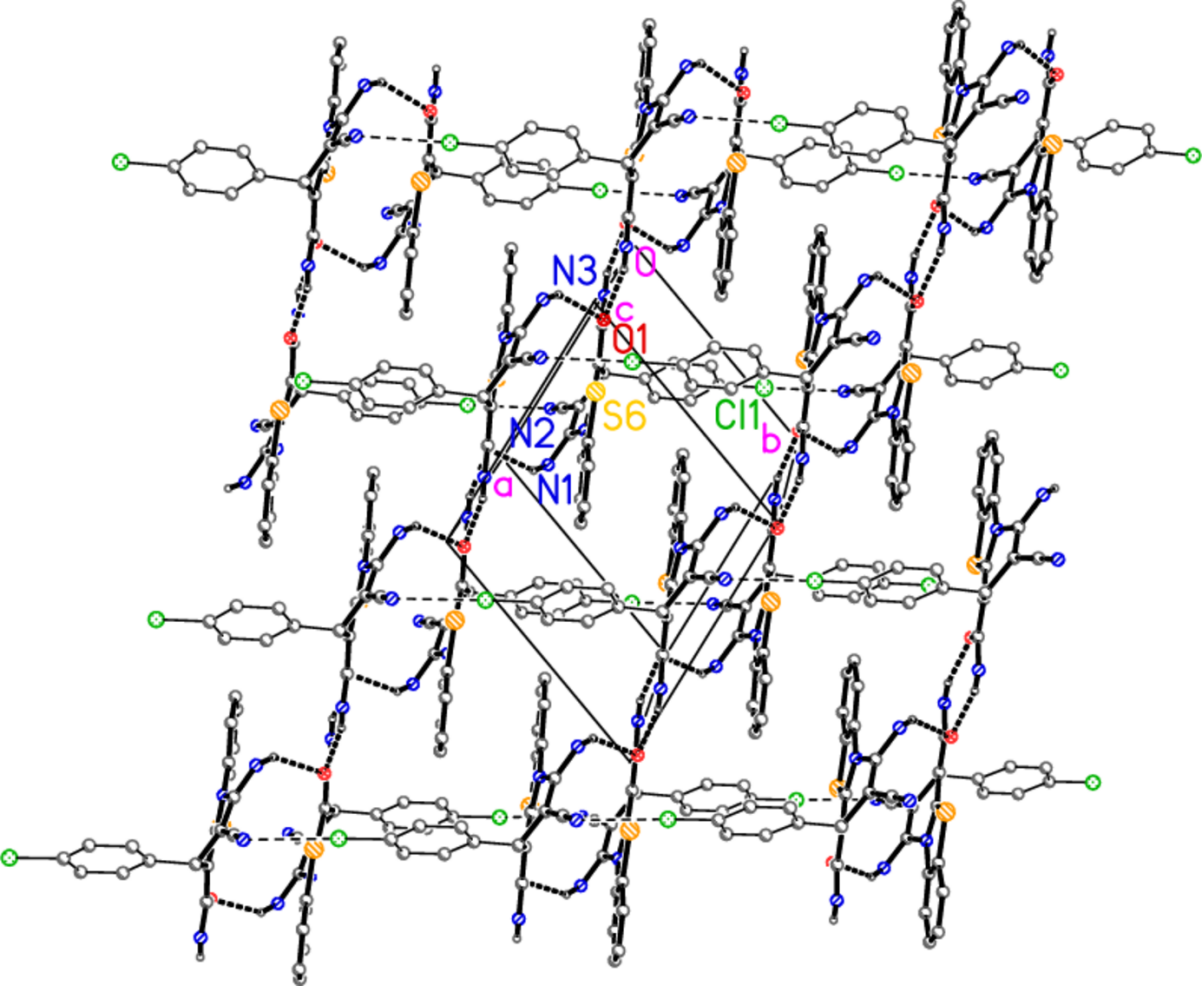
Packing diagram of compound **2** viewed perpendicular to the *ab* plane. Hydrogen bonds are indicated by thick and halogen bonds by thin dashed lines. Hydrogen atoms not involved in hydrogen bonds are omitted for clarity.

The packing is three-dimensional, but can be analysed as two inter­connected layer structures. The first, parallel to the *bc* plane, involves the hydrogen bonds from H01, H02 and H04 (Fig. 4 ▸). Ribbons parallel to the *c* axis are prominent, and these are crosslinked parallel to the *b* axis by the inter­actions H02⋯Cl1. The second and more complex (thicker) layer is parallel to the *ab* plane and involves the hydrogen bonds from H01 and H03 together with the N⋯Cl halogen bonds (Fig. 5 ▸). Ribbons of mol­ecules parallel to [1
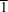
0] (horizontal in Fig. 5 ▸) are prominent; these are linked by the contacts H03⋯O1, which are however difficult to recognize in Fig. 5 ▸ because the inversion-symmetric hydrogen-bond systems are viewed approximately edge-on (they are clearer on the right-hand edge of Fig. 5 ▸).

**Figure 6:**
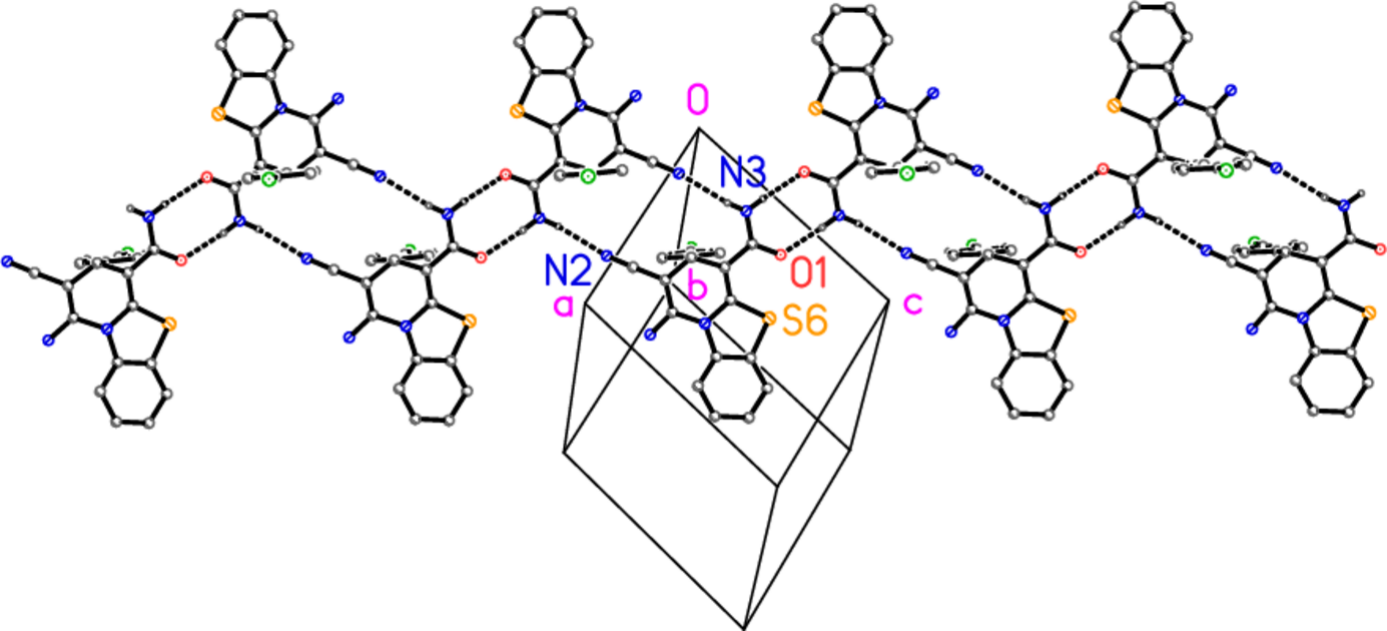
Packing diagram of compound **2** showing a ribbon generated by the hydrogen bonds H04⋯N2 and H03⋯N1 (indicated by dashed lines). The view direction is perpendicular to (1
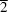
1).

We incorporated three different contacts in both Figs. 4 ▸ and 5 ▸. A referee has correctly pointed out that this comes at the cost of some loss of clarity, and that a much more striking motif comes from the two hydrogen bonds H04⋯N2 and H03⋯O1. The ribbon thus generated is shown in Fig. 6 ▸; it runs parallel to [10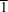]. Neighbouring ribbons are related by the vector [111] (amongst others) and the ribbons thus lie in planes parallel to (1
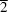
1).

## 4. Database survey

**Figure 7:**
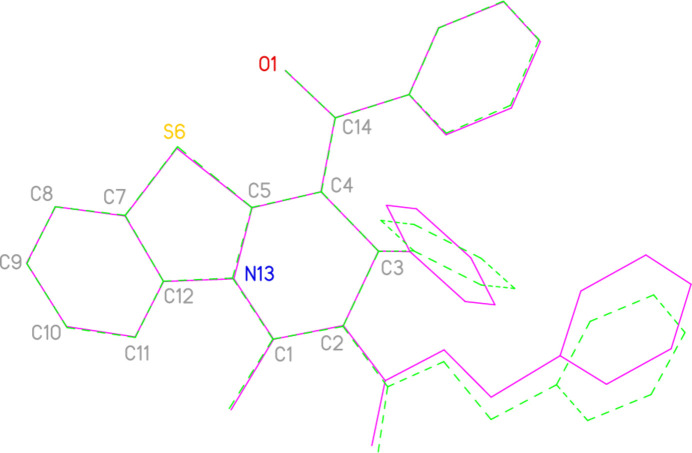
Least-squares fit of the two mol­ecules of **3** (Chauhan & Kumara Swamy, 2024 ▸), renumbered to be consistent with the numbering of **2**. The fitted atoms are labelled.

**Figure 8:**
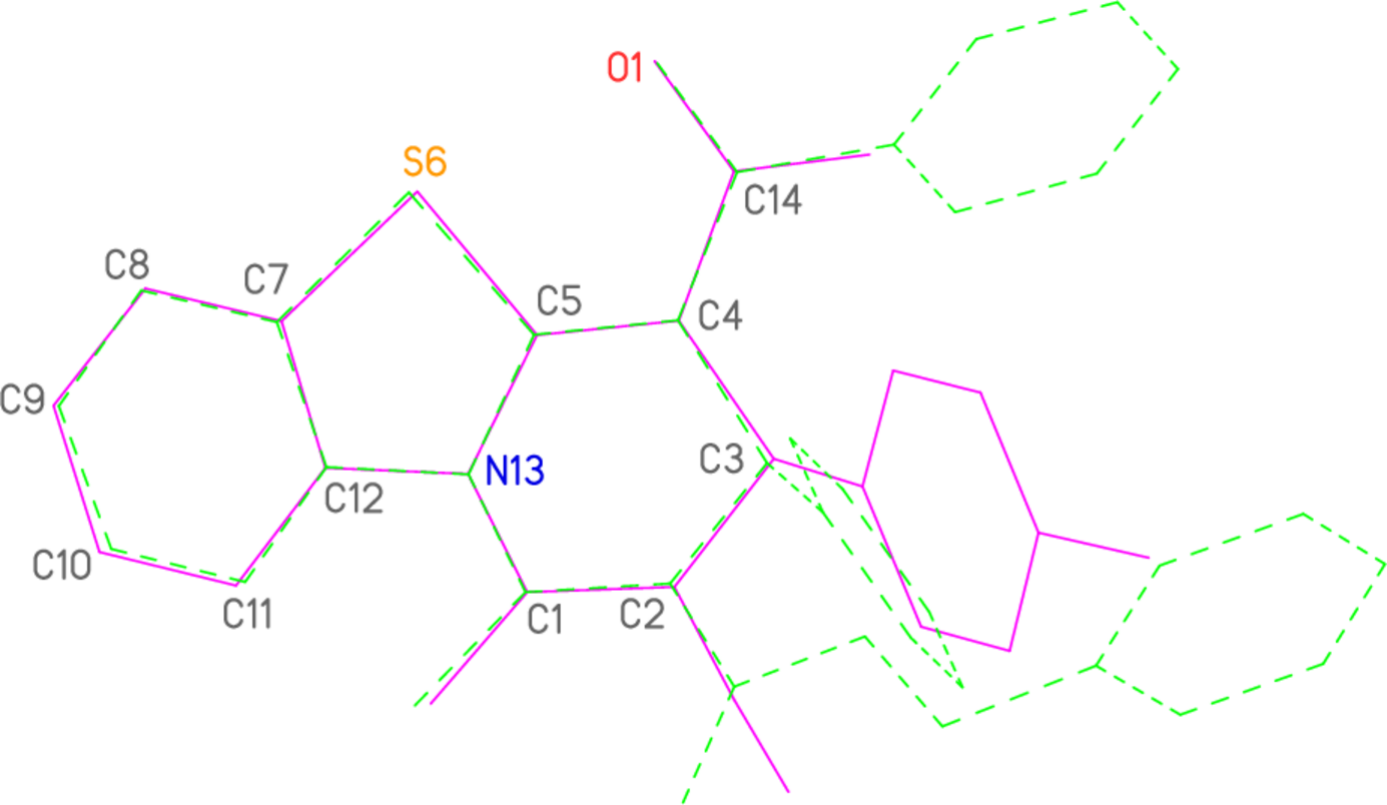
Least-squares fit of **2** (full bonds, purple) to one mol­ecule of **3** (dashed bonds, green). The fitted atoms are labelled.

The search employed the routine ConQuest (Bruno *et al.*, 2002 ▸), part of Version 5.46 of the Cambridge Database (Groom *et al.*, 2016 ▸). Only one structure containing the same tricyclic ring system as that in **2** was found, namely benzyl 4-benzoyl-1-methyl-3-phenyl-3*H*-benzo[4,5]thia­zolo-[3,2-*a*]pyridine-2-carboxyl­ate **3** (Chauhan & Kumara Swamy, 2024 ▸; refcode GOYRAR). This structure involves two independent mol­ecules, which are however closely similar to each other except for ring orientations of the substituents (Fig. 7 ▸; r.m.s. deviation of fitted atoms = 0.029 Å). A similar fit of one mol­ecule of **3** to the mol­ecule of **2** (Fig. 8 ▸) gave an r.m.s deviation of 0.118 Å. There are significant differences between the pyridinic rings C1/C2/C3/C4/C5/N13, *e.g.* the bond length C1—N13, which is 1.3970 (6) / 1.420 (3) / 1.420 (3) Å for **2** and the two mol­ecules in the structure of **3**, in that order, and the ring torsion angles (starting with the bond C5—N13 and moving clockwise, these are −20/−28/−26, 26/28/29, 1/9/5, −30/−43/−37, 36/43/39 and −23/−11/−11, rounded to the nearest degree, for **2** and the two mol­ecules of **3**, in that order).

Similar, but not identical, ring systems were reported in the structures of 2-(1-amino-2-cyano-3-oxo-3*H*-pyrido[2,1-*b*][1,3]benzo­thia­zol-4-yl)-2,3,3-tri­methyl­cyclo­propane-1,1-dicarbo­nitrile methanol solvate (ROPSOH, Rémond *et al.*, 2019 ▸) and 1-amino-2-(1,3-benzo­thia­zol-2-yl)-3*H*-pyrido[2,1-*b*][1,3]ben­zo­thia­zol-3-iminium chloride methanol solvate (REZVUQ; Chen *et al.*, 2018 ▸), both of which have exocyclic double bonds at the atom corresponding to C3 of **2**; and also tetra­methyl 4*aH*-pyrido[2,1-*b*][1,3]benzo­thia­zole-2,3,4,4*a*-tetra­carboxyl­ate and tetra­methyl 1*H*-pyrido[2,1-*b*][1,3]benzo­thia­zole-1,2,3,4-tetra­carboxyl­ate (VIZPIH and VIZPON; Li *et al.*, 2023 ▸) and 5-imino-2,2-dimethyl-1-methyl­idene-1,2-di­hydro-5*H*-furo[3′,2′:3,4]pyrido[2,1-*b*][1,3]benzo­thia­zole-4-carbo­nitrile (ROPQAR; Rémond *et al.*, 2019 ▸), in which the atoms corres­ponding to C5 in **2** bear an additional substituent and the double bond positions correspond to C1—C2 and C3—C4 of **2**.

## 5. Synthesis and crystallization

Equimolar amounts of 2-(1,3-benzo­thia­zol-2-yl)-3-(4-chloro­phen­yl) prop-2-enamide (**1**) (3.15 g, 1 mmol) and malono­nitrile (0.66 g, 1 mmol) were placed in a reaction flask and dissolved in 50 mL dry EtOH. A few drops of piperidine were added and the reaction mixture was heated to reflux for 5 h with stirring. After completing the reaction, the mixture was cooled to room temperature; the solid thus formed was filtered off and dried under vacuum. The product (**2**) was recrystallized from DMF and dried at room temperature.

Pale-yellow crystals, yield 80%, m.p. 578–580 K. IR (KBr): ν (cm^−1^) 3423, 3394 (NH_2_), 3156 (CH aromatic), 2184 (CN), 1644 (C=O); ^1^H-NMR (400 MHz, DMSO-*d*_6_): δ = 4.85 (*s*, 1H, pyridine-H), 6.43 (*s*, 2H, NH_2_), 7.16–7.29 (*m*, 6H, Ar-H, NH_2_), 7.36 (*d*, 2H, *J* = 8.4 Hz, Ar-H), 7.63 (*d*, 1H, *J* = 7.52 Hz, Ar-H), 7.74 (*d*, 1H, *J* = 8.24 Hz, Ar-H) ppm. ^13^C-NMR (100 MHz, DMSO-*d*_6_): δ = 19.02, 56.53, 56.19, 99.53, 116.84, 120.91, 122.77, 124.61, 126.17, 127.88, 129.45, 136.29, 146.93, 148.43, 152.01, 152.03, 167.47 ppm. Analysis calculated for C_19_H_13_ClN_4_OS (380.05): C 59.92, H 3.44, N 14.71, S 8.42. Found: C 60.09, H 2.92, N 14.90, S 8.24%.

## 6. Refinement

Crystal data, data collection and structure refinement details are summarized in Table 3 ▸. The hydrogen atoms of the NH_2_ groups were refined freely. Other hydrogen atoms were included using a riding model starting from calculated positions (C—H_methine_ = 1.00, C—H_arom_ = 0.95 Å). The *U*(H) values were fixed at 1.2 × *U*_eq_ of the parent carbon atoms.

The program *checkCIF* reported a problem with badly-fitting reflections at the level ALERT B: ‘Number of (Iobs-Icalc)/Sigma(W) > 10 Outliers. . 2’. In our experience, this is not unusual for organic structures measured to high diffraction angles. Omitting the five worst reflections in fact led (after an appropriate change of the weighting scheme) to a slight *increase* in *wR*2, so they were retained.

## Supplementary Material

Crystal structure: contains datablock(s) I, global. DOI: 10.1107/S2056989025001562/yz2064sup1.cif

Structure factors: contains datablock(s) I. DOI: 10.1107/S2056989025001562/yz2064Isup2.hkl

Supporting information file. DOI: 10.1107/S2056989025001562/yz2064Isup3.cml

CCDC reference: 2425470

Additional supporting information:  crystallographic information; 3D view; checkCIF report

## Figures and Tables

**Table 1:** Selected geometric parameters (Å, °)

C1—N1	1.3663 (6)	C5—N13	1.3998 (6)
C1—C2	1.3684 (6)	C5—S6	1.7464 (4)
C1—N13	1.3970 (6)	S6—C7	1.7523 (5)
C2—C15	1.4125 (6)	C12—N13	1.4168 (6)
C2—C3	1.5184 (6)	C14—O1	1.2567 (6)
C3—C4	1.5105 (6)	C14—N3	1.3422 (6)
C4—C5	1.3597 (6)	C15—N2	1.1627 (6)
			
C4—C5—S6	126.72 (3)	C1—N13—C5	117.93 (4)
C5—S6—C7	90.51 (2)	C1—N13—C12	126.47 (4)
C8—C7—S6	125.87 (4)	C5—N13—C12	113.70 (4)
C11—C12—N13	128.37 (4)		
			
N13—C1—C2—C3	1.19 (6)	C14—C4—C5—S6	−7.60 (6)
C1—C2—C3—C4	−30.53 (6)	C2—C1—N13—C5	25.76 (6)
C2—C3—C4—C5	35.97 (5)	C4—C5—N13—C1	−19.82 (6)
C3—C4—C5—N13	−13.42 (6)	C5—C4—C14—O1	6.00 (7)

**Table 2:** Hydrogen-bond geometry (Å, °)

*D*—H⋯*A*	*D*—H	H⋯*A*	*D*⋯*A*	*D*—H⋯*A*
N1—H01⋯O1^i^	0.845 (11)	2.391 (11)	3.1341 (6)	147.2 (10)
N1—H02⋯Cl1^ii^	0.906 (13)	2.755 (13)	3.4516 (5)	134.6 (10)
N3—H03⋯O1^iii^	0.837 (13)	2.016 (13)	2.8336 (6)	165.3 (12)
N3—H04⋯N2^iv^	0.834 (12)	2.287 (13)	3.1109 (6)	169.9 (12)
C23—H23⋯S6^v^	0.95	2.90	3.7631 (6)	152

Symmetry codes: (i) 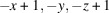; (ii) 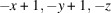; (iii) 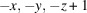; (iv) 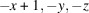; (v) 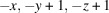.

**Table 3:** Experimental details

Crystal data	
Chemical formula	C_19_H_13_ClN_4_OS
*M*_r_	380.84
Crystal system, space group	Triclinic, *P* 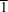
Temperature (K)	100
*a*, *b*, *c* (Å)	9.12784 (17), 9.29662 (17), 10.85172 (19)
α, β, γ (°)	83.0139 (14), 73.4998 (16), 71.4982 (16)
*V* (Å^3^)	836.80 (3)
*Z*	2
Radiation type	Mo *K*α
μ (mm^−1^)	0.37
Crystal size (mm)	0.15 × 0.15 × 0.12

Data collection	
Diffractometer	XtaLAB Synergy
Absorption correction	Multi-scan (*CrysAlis PRO*; Rigaku OD, 2023 ▸)
*T*_min_, *T*_max_	0.953, 1.000
No. of measured, independent and observed [*I* > 2σ(*I*)] reflections	138480, 13768, 11364
*R*_int_	0.041
θ values (°)	θ_max_ = 45.0, θ_min_ = 2.3
(sin θ/λ)_max_ (Å^−1^)	0.996

Refinement	
*R*[*F*^2^ > 2σ(*F*^2^)], *wR*(*F*^2^), *S*	0.029, 0.092, 1.05
No. of reflections	13768
No. of parameters	251
H-atom treatment	H atoms treated by a mixture of independent and constrained refinement
Δρ_max_, Δρ_min_ (e Å^−3^)	0.79, −0.43

Computer programs: *CrysAlis PRO* (Rigaku OD, 2023 ▸), *SHELXT* (Sheldrick, 2015*a* ▸), *SHELXL2019/3* (Sheldrick, 2015*b* ▸) and *XP* (Bruker, 1998 ▸), *publCIF* (Westrip, 2010 ▸).

## supplementary crystallographic information

### 1-Amino-3-(4-chlorophenyl)-2-cyano-3H-benzo[4,5]thiazolo[3,2-a]pyridine-4-carboxamide Crystal data

**Table:**

C_19_H_13_ClN_4_OS	*Z* = 2
*M**_r_* = 380.84	*F*(000) = 392
Triclinic, *P*1	*D*_x_ = 1.511 Mg m^−^^3^
*a* = 9.12784 (17) Å	Mo *K*α radiation, λ = 0.71073 Å
*b* = 9.29662 (17) Å	Cell parameters from 73214 reflections
*c* = 10.85172 (19) Å	θ = 2.3–45.1°
α = 83.0139 (14)°	µ = 0.37 mm^−^^1^
β = 73.4998 (16)°	*T* = 100 K
γ = 71.4982 (16)°	Block, pale yellow
*V* = 836.80 (3) Å^3^	0.15 × 0.15 × 0.12 mm

### 1-Amino-3-(4-chlorophenyl)-2-cyano-3H-benzo[4,5]thiazolo[3,2-a]pyridine-4-carboxamide Data collection

**Table:**

XtaLAB Synergy diffractometer	13768 independent reflections
Radiation source: micro-focus sealed X-ray tube, PhotonJet (Mo) X-ray Source	11364 reflections with *I* > 2σ(*I*)
Mirror monochromator	*R*_int_ = 0.041
Detector resolution: 10.0000 pixels mm^-1^	θ_max_ = 45.0°, θ_min_ = 2.3°
ω scans	*h* = −18→18
Absorption correction: multi-scan (CrysAlisPro; Rigaku OD, 2023)	*k* = −18→18
*T*_min_ = 0.953, *T*_max_ = 1.000	*l* = −21→21
138480 measured reflections	

### 1-Amino-3-(4-chlorophenyl)-2-cyano-3H-benzo[4,5]thiazolo[3,2-a]pyridine-4-carboxamide Refinement

**Table:**

Refinement on *F*^2^	Primary atom site location: dual
Least-squares matrix: full	Hydrogen site location: mixed
*R*[*F*^2^ > 2σ(*F*^2^)] = 0.029	H atoms treated by a mixture of independent and constrained refinement
*wR*(*F*^2^) = 0.092	*w* = 1/[σ^2^(*F*_o_^2^) + (0.0528*P*)^2^ + 0.0873*P*] where *P* = (*F*_o_^2^ + 2*F*_c_^2^)/3
*S* = 1.05	(Δ/σ)_max_ = 0.002
13768 reflections	Δρ_max_ = 0.79 e Å^−^^3^
251 parameters	Δρ_min_ = −0.43 e Å^−^^3^
0 restraints	

### 1-Amino-3-(4-chlorophenyl)-2-cyano-3H-benzo[4,5]thiazolo[3,2-a]pyridine-4-carboxamide Fractional atomic coordinates and isotropic or equivalent isotropic displacement parameters (Å^2^)

**Table:**

	*x*	*y*	*z*	*U*_iso_*/*U*_eq_	
C1	0.62863 (5)	0.18304 (5)	0.26384 (4)	0.00979 (6)	
C2	0.55764 (5)	0.15435 (5)	0.17797 (4)	0.00975 (6)	
C3	0.38005 (5)	0.17027 (5)	0.21294 (4)	0.00936 (6)	
H3	0.366407	0.090592	0.166731	0.011*	
C4	0.32786 (5)	0.13443 (5)	0.35529 (4)	0.00979 (6)	
C5	0.39633 (5)	0.17806 (5)	0.43467 (4)	0.00967 (6)	
S6	0.33319 (2)	0.18032 (2)	0.60235 (2)	0.01162 (2)	
C7	0.48320 (6)	0.25861 (5)	0.60550 (4)	0.01302 (7)	
C8	0.50543 (8)	0.30447 (7)	0.71456 (5)	0.01844 (9)	
H8	0.440305	0.289424	0.797584	0.022*	
C9	0.62517 (9)	0.37289 (7)	0.69938 (6)	0.02188 (10)	
H9	0.643736	0.403125	0.772723	0.026*	
C10	0.71786 (8)	0.39714 (7)	0.57686 (6)	0.02012 (9)	
H10	0.797670	0.445775	0.567855	0.024*	
C11	0.69604 (7)	0.35157 (6)	0.46715 (5)	0.01556 (8)	
H11	0.758821	0.369660	0.383928	0.019*	
C12	0.57950 (6)	0.27875 (5)	0.48314 (4)	0.01175 (6)	
N13	0.53378 (5)	0.22529 (5)	0.38728 (4)	0.01024 (5)	
C14	0.19973 (5)	0.06575 (5)	0.41360 (4)	0.01091 (6)	
C15	0.65300 (5)	0.10088 (5)	0.05570 (4)	0.01129 (6)	
C21	0.27580 (5)	0.32411 (5)	0.17334 (4)	0.01002 (6)	
C22	0.12148 (6)	0.38940 (6)	0.24858 (5)	0.01569 (8)	
H22	0.082993	0.340524	0.327728	0.019*	
C23	0.02267 (6)	0.52478 (6)	0.21005 (5)	0.01809 (9)	
H23	−0.082033	0.568194	0.262528	0.022*	
C24	0.07886 (6)	0.59555 (6)	0.09417 (5)	0.01406 (7)	
C25	0.23230 (6)	0.53403 (6)	0.01705 (5)	0.01415 (7)	
H25	0.270135	0.583256	−0.062092	0.017*	
C26	0.32980 (6)	0.39891 (5)	0.05771 (4)	0.01231 (7)	
H26	0.435145	0.356780	0.005776	0.015*	
N1	0.78675 (5)	0.17302 (5)	0.23985 (4)	0.01353 (6)	
H01	0.8258 (14)	0.1355 (13)	0.3026 (11)	0.023 (3)*	
H02	0.8437 (16)	0.1369 (14)	0.1610 (12)	0.034 (3)*	
N2	0.72660 (6)	0.05978 (6)	−0.04687 (4)	0.01580 (7)	
N3	0.13807 (5)	0.01158 (6)	0.33732 (4)	0.01399 (6)	
H03	0.0645 (15)	−0.0260 (14)	0.3758 (12)	0.031 (3)*	
H04	0.1759 (15)	0.0036 (14)	0.2581 (12)	0.029 (3)*	
Cl1	−0.04686 (2)	0.76356 (2)	0.04690 (2)	0.01904 (3)	
O1	0.14777 (5)	0.06089 (5)	0.53392 (3)	0.01460 (6)	

### 1-Amino-3-(4-chlorophenyl)-2-cyano-3H-benzo[4,5]thiazolo[3,2-a]pyridine-4-carboxamide Atomic displacement parameters (Å^2^)

**Table:**

	*U*^11^	*U*^22^	*U*^33^	*U*^12^	*U*^13^	*U*^23^
C1	0.00992 (14)	0.01110 (14)	0.00817 (13)	−0.00344 (11)	−0.00189 (11)	0.00008 (11)
C2	0.00965 (13)	0.01188 (14)	0.00725 (13)	−0.00326 (11)	−0.00126 (10)	−0.00066 (11)
C3	0.00985 (13)	0.01111 (14)	0.00723 (13)	−0.00380 (11)	−0.00155 (10)	−0.00048 (10)
C4	0.01045 (14)	0.01187 (14)	0.00733 (13)	−0.00455 (11)	−0.00160 (11)	0.00026 (11)
C5	0.01058 (14)	0.01073 (14)	0.00727 (13)	−0.00326 (11)	−0.00151 (10)	−0.00036 (10)
S6	0.01369 (5)	0.01322 (4)	0.00677 (4)	−0.00345 (3)	−0.00127 (3)	−0.00071 (3)
C7	0.01710 (18)	0.01301 (15)	0.00945 (14)	−0.00394 (13)	−0.00428 (13)	−0.00177 (12)
C8	0.0267 (2)	0.0196 (2)	0.01145 (17)	−0.00738 (18)	−0.00704 (16)	−0.00321 (15)
C9	0.0317 (3)	0.0220 (2)	0.0178 (2)	−0.0100 (2)	−0.0117 (2)	−0.00437 (17)
C10	0.0265 (3)	0.0193 (2)	0.0211 (2)	−0.01052 (19)	−0.01112 (19)	−0.00304 (17)
C11	0.01864 (19)	0.01591 (18)	0.01571 (18)	−0.00815 (15)	−0.00618 (15)	−0.00159 (14)
C12	0.01476 (16)	0.01154 (14)	0.01035 (14)	−0.00424 (12)	−0.00459 (12)	−0.00149 (11)
N13	0.01129 (13)	0.01244 (13)	0.00781 (12)	−0.00481 (10)	−0.00186 (10)	−0.00145 (10)
C14	0.01048 (14)	0.01328 (15)	0.00875 (14)	−0.00461 (12)	−0.00140 (11)	0.00065 (11)
C15	0.01112 (15)	0.01340 (15)	0.00857 (14)	−0.00324 (12)	−0.00156 (11)	−0.00097 (11)
C21	0.01000 (14)	0.01172 (14)	0.00826 (13)	−0.00336 (11)	−0.00222 (11)	−0.00002 (11)
C22	0.01240 (16)	0.01564 (17)	0.01317 (17)	−0.00125 (13)	0.00101 (13)	0.00295 (14)
C23	0.01289 (17)	0.01701 (19)	0.01685 (19)	0.00025 (14)	0.00087 (14)	0.00324 (15)
C24	0.01290 (16)	0.01323 (16)	0.01366 (17)	−0.00141 (13)	−0.00331 (13)	0.00137 (13)
C25	0.01363 (16)	0.01496 (17)	0.01114 (15)	−0.00240 (13)	−0.00235 (13)	0.00258 (13)
C26	0.01138 (15)	0.01422 (16)	0.00922 (14)	−0.00260 (12)	−0.00144 (12)	0.00120 (12)
N1	0.00978 (13)	0.01948 (17)	0.01153 (14)	−0.00498 (12)	−0.00243 (11)	−0.00033 (12)
N2	0.01548 (16)	0.02044 (18)	0.00983 (14)	−0.00432 (13)	−0.00068 (12)	−0.00344 (12)
N3	0.01429 (15)	0.02009 (17)	0.01006 (13)	−0.01010 (13)	−0.00136 (11)	−0.00046 (12)
Cl1	0.01564 (5)	0.01623 (5)	0.01936 (5)	0.00067 (4)	−0.00360 (4)	0.00431 (4)
O1	0.01479 (14)	0.02202 (16)	0.00799 (12)	−0.00936 (12)	−0.00088 (10)	0.00136 (11)

### 1-Amino-3-(4-chlorophenyl)-2-cyano-3H-benzo[4,5]thiazolo[3,2-a]pyridine-4-carboxamide Geometric parameters (Å, º)

**Table:**

C1—N1	1.3663 (6)	C11—C12	1.3941 (7)
C1—C2	1.3684 (6)	C11—H11	0.9500
C1—N13	1.3970 (6)	C12—N13	1.4168 (6)
C2—C15	1.4125 (6)	C14—O1	1.2567 (6)
C2—C3	1.5184 (6)	C14—N3	1.3422 (6)
C3—C4	1.5105 (6)	C15—N2	1.1627 (6)
C3—C21	1.5345 (6)	C21—C22	1.3942 (7)
C3—H3	1.0000	C21—C26	1.3982 (6)
C4—C5	1.3597 (6)	C22—C23	1.3922 (7)
C4—C14	1.4599 (6)	C22—H22	0.9500
C5—N13	1.3998 (6)	C23—C24	1.3870 (7)
C5—S6	1.7464 (4)	C23—H23	0.9500
S6—C7	1.7523 (5)	C24—C25	1.3897 (7)
C7—C8	1.3921 (7)	C24—Cl1	1.7382 (5)
C7—C12	1.3971 (7)	C25—C26	1.3940 (7)
C8—C9	1.3928 (9)	C25—H25	0.9500
C8—H8	0.9500	C26—H26	0.9500
C9—C10	1.3941 (10)	N1—H01	0.845 (11)
C9—H9	0.9500	N1—H02	0.906 (13)
C10—C11	1.3955 (7)	N3—H03	0.837 (13)
C10—H10	0.9500	N3—H04	0.834 (12)
			
N1—C1—C2	125.51 (4)	C11—C12—C7	120.44 (4)
N1—C1—N13	116.24 (4)	C11—C12—N13	128.37 (4)
C2—C1—N13	118.25 (4)	C7—C12—N13	111.08 (4)
C1—C2—C15	119.32 (4)	C1—N13—C5	117.93 (4)
C1—C2—C3	121.78 (4)	C1—N13—C12	126.47 (4)
C15—C2—C3	118.82 (4)	C5—N13—C12	113.70 (4)
C4—C3—C2	107.98 (3)	O1—C14—N3	121.44 (4)
C4—C3—C21	112.20 (3)	O1—C14—C4	119.33 (4)
C2—C3—C21	114.45 (4)	N3—C14—C4	119.22 (4)
C4—C3—H3	107.3	N2—C15—C2	177.40 (5)
C2—C3—H3	107.3	C22—C21—C26	118.16 (4)
C21—C3—H3	107.3	C22—C21—C3	120.83 (4)
C5—C4—C14	117.99 (4)	C26—C21—C3	120.93 (4)
C5—C4—C3	118.16 (4)	C23—C22—C21	121.32 (5)
C14—C4—C3	123.69 (4)	C23—C22—H22	119.3
C4—C5—N13	121.84 (4)	C21—C22—H22	119.3
C4—C5—S6	126.72 (3)	C24—C23—C22	119.23 (5)
N13—C5—S6	111.43 (3)	C24—C23—H23	120.4
C5—S6—C7	90.51 (2)	C22—C23—H23	120.4
C8—C7—C12	121.12 (5)	C23—C24—C25	120.98 (5)
C8—C7—S6	125.87 (4)	C23—C24—Cl1	118.59 (4)
C12—C7—S6	112.95 (3)	C25—C24—Cl1	120.43 (4)
C7—C8—C9	118.59 (5)	C24—C25—C26	118.92 (4)
C7—C8—H8	120.7	C24—C25—H25	120.5
C9—C8—H8	120.7	C26—C25—H25	120.5
C8—C9—C10	120.19 (5)	C25—C26—C21	121.38 (4)
C8—C9—H9	119.9	C25—C26—H26	119.3
C10—C9—H9	119.9	C21—C26—H26	119.3
C9—C10—C11	121.48 (5)	C1—N1—H01	113.3 (8)
C9—C10—H10	119.3	C1—N1—H02	112.1 (8)
C11—C10—H10	119.3	H01—N1—H02	117.5 (11)
C12—C11—C10	118.12 (5)	C14—N3—H03	115.2 (9)
C12—C11—H11	120.9	C14—N3—H04	124.2 (9)
C10—C11—H11	120.9	H03—N3—H04	120.1 (12)
			
N1—C1—C2—C15	4.07 (7)	N1—C1—N13—C5	−153.81 (4)
N13—C1—C2—C15	−175.46 (4)	C2—C1—N13—C5	25.76 (6)
N1—C1—C2—C3	−179.29 (4)	N1—C1—N13—C12	9.45 (7)
N13—C1—C2—C3	1.19 (6)	C2—C1—N13—C12	−170.98 (4)
C1—C2—C3—C4	−30.53 (6)	C4—C5—N13—C1	−19.82 (6)
C15—C2—C3—C4	146.13 (4)	S6—C5—N13—C1	159.01 (3)
C1—C2—C3—C21	95.20 (5)	C4—C5—N13—C12	174.83 (4)
C15—C2—C3—C21	−88.14 (5)	S6—C5—N13—C12	−6.34 (5)
C2—C3—C4—C5	35.97 (5)	C11—C12—N13—C1	25.29 (8)
C21—C3—C4—C5	−91.07 (5)	C7—C12—N13—C1	−158.51 (4)
C2—C3—C4—C14	−148.76 (4)	C11—C12—N13—C5	−170.85 (5)
C21—C3—C4—C14	84.19 (5)	C7—C12—N13—C5	5.35 (6)
C14—C4—C5—N13	171.04 (4)	C5—C4—C14—O1	6.00 (7)
C3—C4—C5—N13	−13.42 (6)	C3—C4—C14—O1	−169.28 (4)
C14—C4—C5—S6	−7.60 (6)	C5—C4—C14—N3	−175.18 (4)
C3—C4—C5—S6	167.94 (3)	C3—C4—C14—N3	9.55 (7)
C4—C5—S6—C7	−176.96 (4)	C4—C3—C21—C22	−22.43 (6)
N13—C5—S6—C7	4.28 (4)	C2—C3—C21—C22	−145.92 (5)
C5—S6—C7—C8	175.90 (5)	C4—C3—C21—C26	160.82 (4)
C5—S6—C7—C12	−1.30 (4)	C2—C3—C21—C26	37.33 (6)
C12—C7—C8—C9	0.71 (8)	C26—C21—C22—C23	0.40 (8)
S6—C7—C8—C9	−176.28 (5)	C3—C21—C22—C23	−176.43 (5)
C7—C8—C9—C10	1.29 (10)	C21—C22—C23—C24	0.31 (9)
C8—C9—C10—C11	−1.26 (10)	C22—C23—C24—C25	−0.61 (9)
C9—C10—C11—C12	−0.79 (9)	C22—C23—C24—Cl1	179.40 (5)
C10—C11—C12—C7	2.78 (8)	C23—C24—C25—C26	0.19 (8)
C10—C11—C12—N13	178.66 (5)	Cl1—C24—C25—C26	−179.82 (4)
C8—C7—C12—C11	−2.79 (8)	C24—C25—C26—C21	0.55 (8)
S6—C7—C12—C11	174.56 (4)	C22—C21—C26—C25	−0.84 (7)
C8—C7—C12—N13	−179.33 (5)	C3—C21—C26—C25	176.00 (4)
S6—C7—C12—N13	−1.99 (5)		

### 1-Amino-3-(4-chlorophenyl)-2-cyano-3H-benzo[4,5]thiazolo[3,2-a]pyridine-4-carboxamide Hydrogen-bond geometry (Å, º)

**Table:**

*D*—H···*A*	*D*—H	H···*A*	*D*···*A*	*D*—H···*A*
N1—H01···O1^i^	0.845 (11)	2.391 (11)	3.1341 (6)	147.2 (10)
N1—H02···Cl1^ii^	0.906 (13)	2.755 (13)	3.4516 (5)	134.6 (10)
N3—H03···O1^iii^	0.837 (13)	2.016 (13)	2.8336 (6)	165.3 (12)
N3—H04···N2^iv^	0.834 (12)	2.287 (13)	3.1109 (6)	169.9 (12)
C23—H23···S6^v^	0.95	2.90	3.7631 (6)	152

Symmetry codes: (i) −*x*+1, −*y*, −*z*+1; (ii) −*x*+1, −*y*+1, −*z*; (iii) −*x*, −*y*, −*z*+1; (iv) −*x*+1, −*y*, −*z*; (v) −*x*, −*y*+1, −*z*+1.
